# The therapeutic effect of controlled reoxygenation on chronic hypoxia-associated brain injury

**DOI:** 10.1242/bio.039370

**Published:** 2019-12-11

**Authors:** Zhenzhen Tu, Xingqin Tan, Shangyingying Li, Jie Cui, Shengfen Tu, Li Jiang

**Affiliations:** 1 Department of Anesthesiology, Children's Hospital of Chongqing Medical University, Ministry of Education Key Laboratory of Child Development and Disorders; National Clinical Research Center for Child Health and Disorders, Chongqing 400016, China; 2 China International Science and Technology Cooperation base of Child development and Critical Disorders, Chongqing 400016, China; 3 Chongqing Key Laboratory of Pediatrics, Chongqing 400016, China; 4 Department of Neurology, Children's Hospital of Chongqing Medical University, Ministry of Education Key Laboratory of Child Development and Disorders; National Clinical Research Center for Child Health and Disorders, Chongqing 400016, China

**Keywords:** Chronic hypoxia, Brain injury, Reoxygenation, Neuroinflammation, Apoptosis, Therapeutic effect

## Abstract

Cardiopulmonary bypass (CPB) is the most general technique applied in congenital heart disease (CHD). However, standard CPB poses a specific pathologic condition for patients during surgery: exposure to reoxygenation. When surgery is performed on cyanotic infants, standard CPB is usually initiated at a high concentration of oxygen without consideration of cytotoxic effects. Controlled reoxygenation is defined as using normoxic CPB with a pump primed to the PO2 (oxygen tension in the blood), which is matched to the patient's preoperative saturation. The aim of this study was to determine whether controlled reoxygenation could avoid standard reoxygenation injury and also to clarify the molecular signaling pathways during hypoxia. We successfully reproduced the abnormal brain observed in mice with chronic hypoxia during early postnatal development – equivalent to the third trimester in human. Mice were treated with standard reoxygenation and controlled reoxygenation after hypoxia for 24 h. We then assessed the brain tissue of these mice. In standard reoxygenation-treated hypoxia mice, the caspase-3-dependent neuronal apoptosis was enhanced by increasing concentration of oxygen. Interestingly, controlled reoxygenation inhibited neuron and glial cell apoptosis through suppressing cleavage of caspase-3 and PARP. We also found that controlled reoxygenation suppressed LCN2 expression and inflammatory cytokine (including TNF-α, IL-6, and CXCL10) production, in which the JAK2/STAT3 signaling pathway might participate. In conclusion, our findings propose the novel therapeutic potential of controlled reoxygenation on CPB during CHD.

## Abstract (precis)

**Summary:** Controlled reoxygenation may provide an effective therapeutic strategy for hypoxia-induced tissue injury via regulation of the JAK2/STAT3 signaling pathway. It will help make better informed clinical treatment decisions for cyanotic infants.

## INTRODUCTION

Cardiopulmonary bypass (CPB) is a predominant technique associated with congenital heart disease (CHD) surgery, especially cyanotic ones. This surgery is known to have greatly reduced mortality rates, but patients often suffer an array of neurological deficits throughout their lives (Sturmer et al., 2018; Marino et al., 2012). Although clinical evidence from neuroimaging studies has indicated CHD is linked with white-matter immaturity during the prenatal period (Ishibashi et al., 2012), postoperative brain injury has been demonstrated to be signiﬁcantly associated with preoperative chronic hypoxia and model CPB (Hogue et al., 2008). Clinically, when surgery is performed on cyanotic infants, standard CPB is usually initiated at high PaO_2_, without consideration of possible cytotoxic effects of hyperoxia. One of the strategies proposed to avoid reoxygenation injury has been the use of controlled reoxygenation using PaO_2_ similar to the patient's preoperative oxygen saturation when starting CPB (Ihnken et al., 1995). It has been shown to ameliorate reoxygenation injury in adult patients and cyanotic pediatric patients (Caputo et al., 2014). However, cellular events secondary to controlled reoxygenation and the impact of controlled reoxygenation in the developing brain remain largely unexplored.

The Janus kinase/signal transducer and activator of transcription (JAK/STAT) signaling pathway is involved in physiological and pathological processes, such as immune responses, hematopoiesis, cellular homeostasis, gliogenesis and reactive astrocytosis (Shuai and Liu, 2003). STAT3, one of seven members of the STAT family of proteins, participates in cellular responses to cytokines and growth factors as transcription factors (Yang et al., 2005; Wang et al., 2007). Activation of STAT3 is due to JAK2-dependent phosphorylation and JAK2-independent phosphorylation (Aggarwal et al., 2009). In the central nervous system (CNS), STAT3 is expressed by astrocytes, neurons and other glial cell types, and the phosphorylation of STAT3 was found markedly increased during hypoxic brain injury (Hristova et al., 2016).

The objective of our study was to determine the effects of controlled reoxygenation on the developing brain in neonatal mice undergoing chronic hypoxia, and then to reveal whether JAK2/STAT3 signaling is related to controlled reoxygenation-induced reduction of reoxygenation injury. The effects were determined by measuring LCN2, JAK2/STAT3 and their downstream targets, including BNIP and caspase-3.

## RESULTS

### Reduction of neurons and glial cells apoptosis under hypoxia-induced brain injury by controlled reoxygenation treatment

**Fig. 1.**
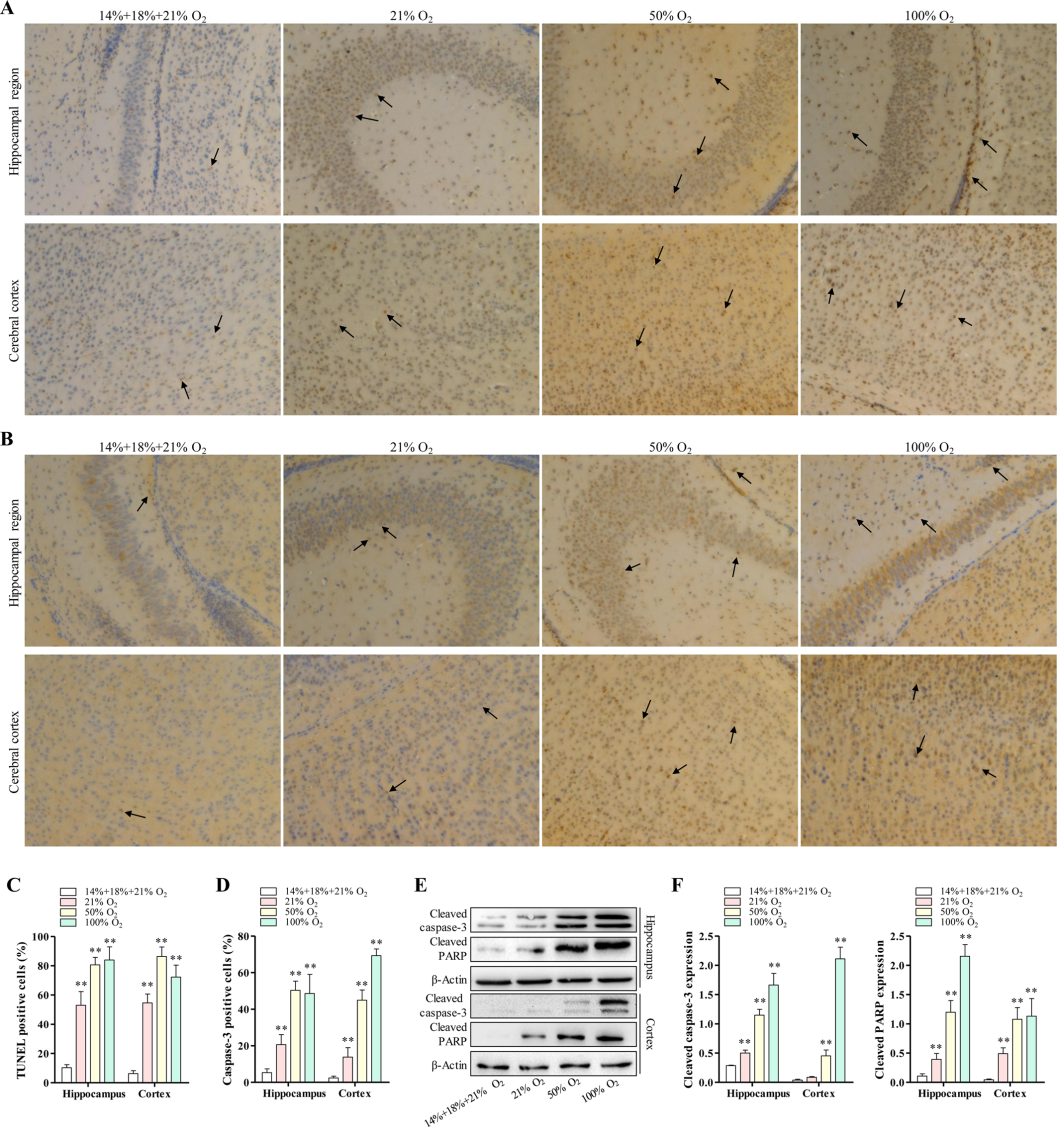
**Controlled reoxygenation reduces hypoxia-induced neuronal apoptosis.** The mice first received hypoxic treatment (10% O_2_) for 8 days, and then reoxygenation treatment (14%+18%+21% O_2_, 21% O_2_, 50% O_2_ and 100% O_2_) for 24 h (*n*=6 mice of each group). (A,C) TUNEL assay for apoptotic cells in hippocampus region and cortex region of reoxygenated mice. Arrows indicate the TUNEL-positive cells. Scale bar: 200 μm. (B,D) Caspase-3 in hippocampus region and cortex region were analyzed by immunohistochemistry staining. Arrows indicate the Caspase-3-positive cells. Scale bar: 200 μm. (E) The expression of cleaved caspase-3 and cleaved PARP in hippocampus region (upper panel) and in cortex region (lower panel) measured with western blotting. (F) Intensities of protein bands standardized to those of β-actin and expressed as relative band intensities. Data represents three independent experiments. Error bars reflect±s.e.m. *P<0.05, **P<0.01.

Occurrence of cell death in hypoxic mice was observed by means of terminal deoxynucleotidyl dUTP nick end labeling (TUNEL) assay. As shown in Fig. 1A and C, the number of apoptotic neurons and glial cells in the hippocampal region and cerebral cortex of mice increased when the concentration of the standard reoxygenation treatment increased. However, the limited apoptotic cells were detected in the controlled reoxygenation-treated hippocampus region and the cortex region of mice (Fig. 1A,C). We further performed immunohistochemistry staining to observe the caspase-3, a cell apoptosis-associated protein, distributed in the hippocampal region and cerebral cortex region. Similarly, increasing the concentration of the standard reoxygenation significantly enhanced the distribution of caspase-3 in both the hippocampal region and cerebral cortex of hypoxic mice (Fig. 1B,D). In contrast, the number of caspase-3 positive cells in mice treated with controlled reoxygenation was lower than that of the mice that underwent standard reoxygenation (Fig. 1B,D).

Activation of caspase-3 plays an important role in cell apoptosis (Salvesen, 2002). Next, we investigated the expression of the cleaved caspase-3 and poly (ADP-ribose) polymerase (PARP) by western blotting (Fig. 1E,F). We found that cleaved caspase-3 and cleaved PARP in both the hippocampus and cortex regions were significantly increased by the treatment with standard reoxygenation in a dose-dependent manner (Fig. 1E,F). In the case of the controlled reoxygenation-treated hippocampus region and cortex region of mice, we observed significantly lower expression of cleaved PARP and cleaved caspase-3 in the cortex region. Limited cleaved caspase-3 and cleaved PARP bands were detected in the hippocampus region. These data suggest that controlled reoxygenation is capable of reducing hypoxia-induced cell apoptosis, in which inhibition of caspase-3 and/or PARP cleavage may play a part role.

### The anti-inflammation effect of controlled reoxygenation on hypoxia-induced brain injury mice

**Fig. 2.**
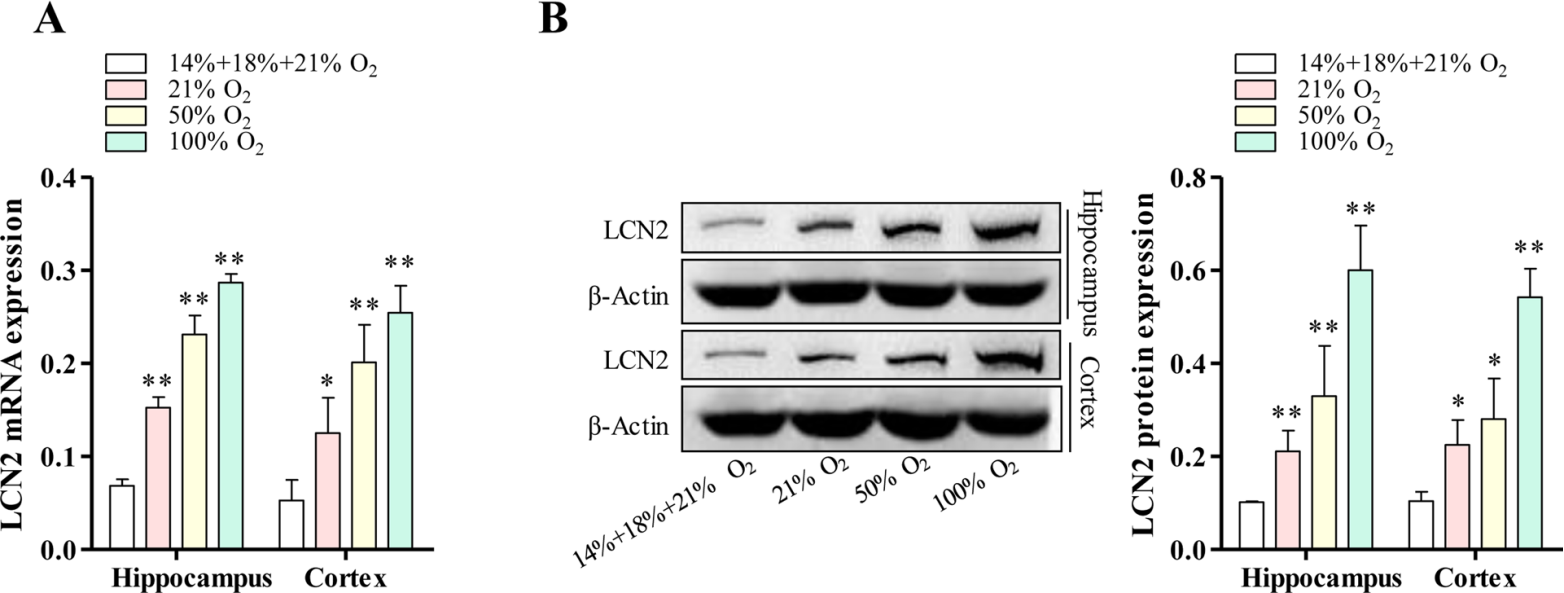
**Controlled reoxygenation reduces the expression of LCN2 in both gene and protein levels.** (A) Quantitative PCR analysis of LCN2 gene transcript. These data were normalized with an amount of β-actin as an internal control. (B) The expression of LCN2 in the hippocampus region (upper panel) and in the cortex region (lower panel) was detected by western blotting. Intensities of protein bands were standardized to those of β-actin and expressed as relative band intensities. Data represents three independent experiments. Error bars reflect±s.e.m. *P<0.05, **P<0.01.

It has been reported that lipocalin-2 (LCN2) plays a critical role in several pathological organ conditions, including liver injury, renal damage, brain injury as well as lung infection (Suk, 2016). In our experiments, the expression of LCN2 at both the gene level and protein level was enhanced by standard reoxygenation, but it was strongly decreased by controlled reoxygenation in both the hippocampus region and cortex region (Fig. 2A,B).

**Fig. 3.**
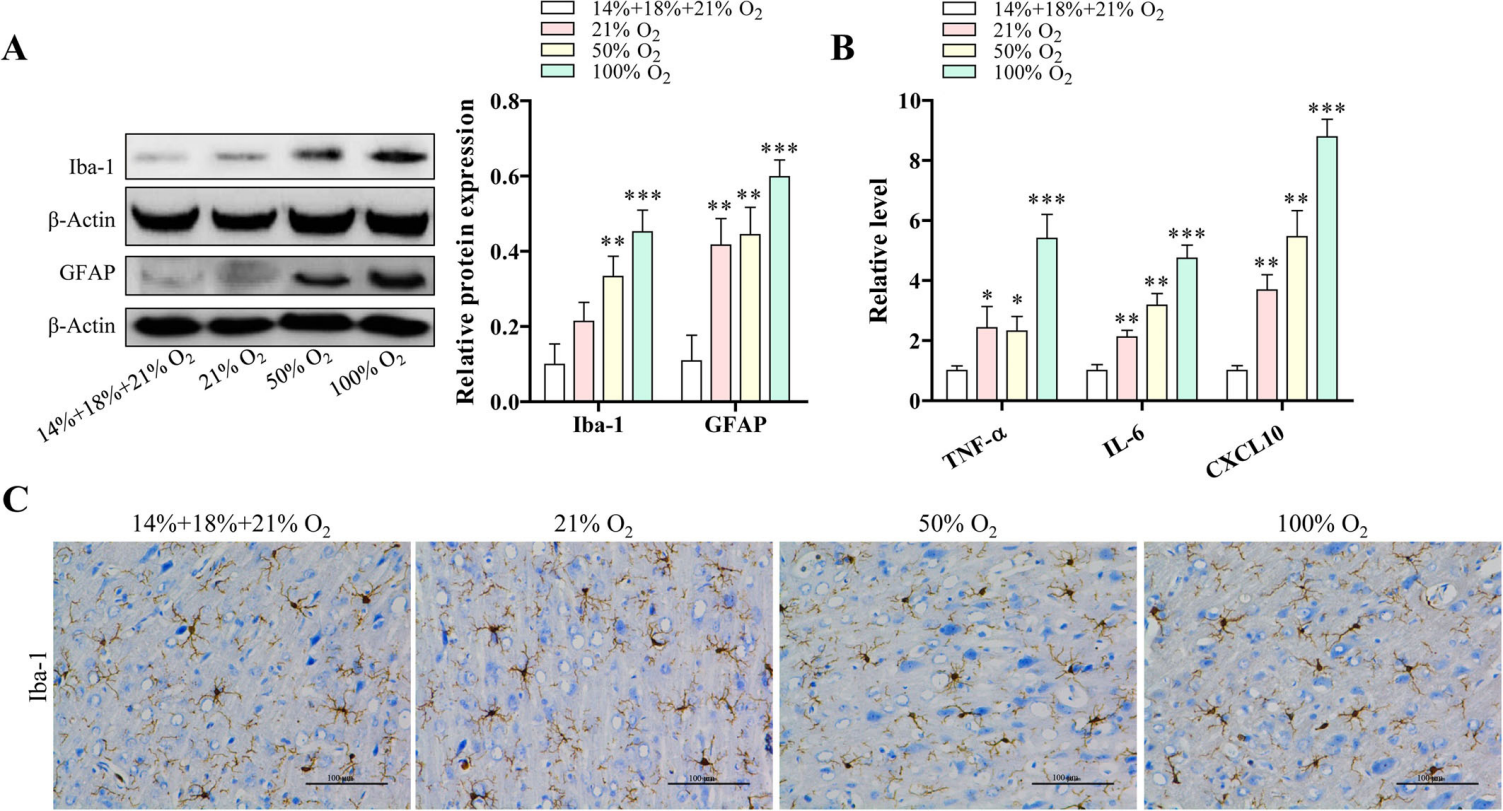
**Controlled reoxygenation reduces the neuroinflammation in hypoxia-induced brain injury mice.** (A) The expression of Iba-1 and GFAP was analyzed by western blotting. Intensities of protein bands were standardized to those of β-actin and expressed as relative band intensities. (B) Cytokine TNF-α, IL-6, as well as chemokine CXCL10 secreted from each group of mice were measured by ELISA. (C) Iba-1 was observed by immunohistochemistry staining. Scale bars: 100 μm. Data represents three independent experiments. Error bars reflect±s.e.m. *P<0.05, **P<0.01.

LCN2 expression is highly regulated in astrocytes under the condition of diverse brain injury, which further causes neuroinflammation (Suk, 2016). In order to clarify whether controlled reoxygenation can ameliorate the development of LCN2-induced neuroinflammation, we examined microglial activation by western blotting for Iba-1 and glial fibrillary acidic protein (GFAP). Consistent with our hypothesis, expression of Iba-1 and GFAP was clearly enhanced by standard reoxygenation in a dose-dependent manner (Fig. 3A). Importantly, compared with standard reoxygenation-treated mice, the immuno-reactive bands of Iba-1 and GFAP were remarkably attenuated in the controlled reoxygenation-treated hippocampus region and cortex region of mice (Fig. 3A). This trend was also confirmed by immunohistochemistry stained with Iba-1 (Fig. 3C). Meanwhile, the effects of standard reoxygenation and controlled reoxygenation on cytokine as well as chemokine were also examined. Our data showed that standard reoxygenation treatment increased secretion of TNF-α, IL-6 and CXCL10 in a dose-dependent manner, but they were significantly decreased in the mice treated by controlled reoxygenation (Fig. 3B). This finding again supported the notion that controlled reoxygenation has an anti-inflammatory effect on hypoxia-associated neuroinflammation.

### Downregulation by controlled reoxygenation treatment of JAK2/STAT3 and AMPK/mTOR signaling pathway in hypoxic-induced brain injury mice

**Fig. 4.**
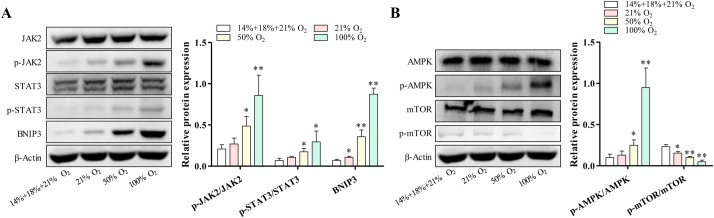
**Regulation of JAK2/STAT3 and AMPK/mTOR signaling pathway by reoxygenation in hypoxia-induced brain injury mice.** (A) The phosphorylation of JAK2 and STAT3 as well as the expression of its downstream molecule BNIP3 were analyzed by western blotting. (B) Autophagy-associated signaling was also observed by western blotting with antibodies against AMPK and mTOR. Intensities of protein bands were standardized to those of β-actin and expressed as relative band intensities. Data represent three independent experiments. Error bars reflect±s.e.m. *P<0.05, **P<0.01.

The JAK2/STAT3 signaling pathway is known to be involved in the pathological processes of CNS insults (Nicolas et al., 2013). We finally investigated whether JAK/STAT3-mediated neuroinflammation directly regulated by oxygenation. As shown in Fig. 4A, standard reoxygenation markedly increased the phosphorylation of JAK2 (p-JAK2) and STAT3 (p-STAT3). However, the expression of p-JAK2 and p-STAT3 was significantly lower the in controlled reoxygenation-treated hippocampus region and cortex region of mice than those treated by standard reoxygenation. These results suggest that the cytoprotective and anti-inflammatory effects of controlled oxygenation on hypoxia-induced brain injury are associated with the JAK2/STAT3 signaling pathway. Interestingly, we observed that bcl-2/E1B 19 kDa interacting protein 3 (BNIP3), an autophagy biomarker, was increased by treatment of standard reoxygenation in a dose-dependent manner. However, the expression of BNIP3 was suppressed by controlled reoxygenation treatment. We expected that autophagy is induced as a scavenger of dead cells by standard reoxygenation during hypoxia-induced brain injury. To prove our hypothesis, the analysis of autophagy marker proteins was performed by western blotting. mTOR is a known critical negative regulator for autophagy that is inactivated by AMPK phosphorylation (Jung et al., 2010). In contrast to the controlled reoxygenation-treated hippocampus region and cortex region of mice, the standard reoxygenation-treated hippocampus region and cortex region of mice showed more expression of phosphorylation of AMPK, lower expression of phosphorylated mTOR (Fig. 4B). These results provide further evidence that controlled reoxygenation decreased cell apoptosis during hypoxia-induced brain injury. In other words, controlled reoxygenation suppresses neuroinflammation and protects neurons from apoptosis, in which the JAK2/STAT3 signaling pathway and its downstream signals probably participate.

## DISCUSSION

In this study, we successfully reproduced the abnormal brain observed in mice with chronic hypoxia during early postnatal development, which is similar to the abnormal brain in cyanotic pediatric patients and is equivalent to the third trimester in human. During CPB, high oxygen levels are the major cause of brain injury (Hogue et al., 2008; Fontes et al., 2014). We have replicated this specific brain condition in the CPB by using mice models and provided evidence for the less detrimental effects of controlled reoxygenation on hypoxia-induced brain injury. For instance, we observed that standard reoxygenation caused caspase-3 associated-neuron and glial cell apoptosis in a dose-dependent manner after mice hypoxia; however, controlled reoxygenation treatment resulted in a significant decrease in hypoxia-induced neurons and glial cells apoptosis via regulation of caspase-3 activation (Fig. 1A–F). In their paper, Caretti et al. demonstrated that chronic hypoxia could induce neuron apoptosis by upregulating p-ERK1/2 and p-p38 activity (Caretti et al., 2008); similarly, Fantacci et al. revealed that both hypoxic brain and myocardial tissue exhibited a strong apoptotic signal (Fantacci et al., 2006). The present study is one of the few studies to explore the effect of the strictly controlled reoxygenation treatment on brain tissues injury induced by chronic hypoxia. Our observation is supported by one previous study that revealed gradual reoxygenation after hypoxia could effectively weaken myocardial injury in infants with cyanosis who undergo cardiac repair with the use of CPB (Corno et al., 1995). These findings may suggest that standard CPB poses a specific pathologic condition which is exposure to standard reoxygenation for patients undergoing cardiac surgery.

It has been demonstrated that LCN2 expression is mainly associated with hypoxia-induced brain injury (Suk, 2016). Consistent with this, our data shows that LCN2 expression was upregulated by standard reoxygenation, which was reduced after treatment of controlled reoxygenation in both the hippocampus region and cortex region of hypoxia mice (Fig. 2A,B). Additionally, some studies described that upregulation of LCN2 expression is mediated through several cytokines or chemokines, such as TNF-α, IL-6, CXCL10, etc., (Nam et al., 2014; Kang et al., 2018). Consistent with this, our present study showed that standard reoxygenation enhanced expression of LCN2 and secretion of cytokines as well as chemokines (Fig. 3). However, the reduction of LCN2 expression was significantly lower under the treatment of controlled reoxygenation compared with the groups treated by standard reoxygenation (Fig. 2). These results indicated that dysregulation of LCN2 may be not only associated with neuroinflammation, but also be responsible for neuronal apoptosis. Accumulating evidence has suggested that, in hypoxia mice, the expression of LCN2 plays a key role in brain inflammation and the controlled reoxygenation is an effective treatment for neuroinflammation and neuronal apoptosis.

It has been reported that Iba1 is specifically expressed in macrophages and microglia, where it is upregulated during the activation of these cells (Ito et al., 1998). There are several kinds of receptor including cytokine receptors, scavenger receptors, chemokine receptors and pattern recognition receptors on the microglia cell membrane, activation of these receptors by inflammatory molecules or pathogens result in a rapid activation of resting microglia (Kierdorf and Prinz, 2013). In this study, we found that upregulation of Iba-1 was induced by standard reoxygenation treatment but not by controlled reoxygenation (Fig. 3A,C). Moreover, inflammatory cytokines and chemokine were enhanced in standard reoxygenation-treated mice, and we did not see the release of cytokines and chemokine in the controlled reoxygenation-treated mice (Fig. 3B). Indeed, there is an enhancement of neuroinflammation in hypoxia mice by standard reoxygenation treatment, meanwhile, neuroinflammation was impaired by controlled reoxygenation treatment. It is noted that not only Iba-1 but also GFAP was also increased by standard reoxygenation treatment in hypoxia mice (Fig. 3A). It suggests that standard reoxygenation treatment induces neuron apoptosis during hypoxic conditions. Collectively, our data indicated that controlled reoxygenation treatment is more beneficial than standard reoxygenation treatment during cyanotic infant surgery.

The JAK2/STAT3 pathway is an important intracellular signal-transduction pathway that relays extracellular signals to nuclei (Kiu and Nicholson, 2012). Activated STAT3 (p-STAT3) is involved in organ protection and cell survival during ischemia/reperfusion injury (Oshima et al., 2005; Boengler, 2011). Kim et al. and Zhao et al. demonstrated that activation of the JAK2/STAT3 pathway can reduce neuronal apoptosis and recover neurological function (Kim et al., 2017; Zhao et al., 2011). However, some studies have shown that inhibition of activation of the JAK2/STAT3 pathway can reduce neonatal hypoxic–ischemic brain injury (Hristova et al., 2016). We found that the beneficial effects of controlled reoxygenation were associated with a reduction of the expression of p-JAK2 and p-STAT3 in the brain (Fig. 4A). These different results may be related to the experimental model, experimental object, experimental conditions and the degree of injury. Considering these data, we hypothesize that the body can play a protective role by activating the JAK2/STAT3 pathway when the brain is injured. In low oxygen level conditions, glucose and amino acids induce AMPK activity and delay neuronal death by apoptosis during hypoxia (Perez-Alvarez et al., 2018). mTOR plays a key role in the apoptosis signaling pathway downstream of AMPK, for instance, by regulating autophagy through mTOR activity. Cellular death is triggered by upregulation of AMPK and deregulation of mTOR. At the same time, autophagy is activated to degrade dysfunctional or unnecessary cellular components through the lysosomes, and increased activity of this pathway has been proven to ameliorate the damage (Perez-Alvarez et al., 2018). We demonstrate that controlled reoxygenation treatment is associated with the reduction of AMPK activity and the enhancement of mTOR activity (Fig. 4B) in brain injury mice compared with standard reoxygenation-treated mice. These results indicated that controlled reoxygenation treatment successfully ameliorates the degree of brain injury, because autophagy might not be activated. Another possible reason for this inhibition of autophagy may be the different mechanism, which is the crosslink between the JAK2/STAT3 signaling pathway and the AMPK/mTOR signaling pathway. Further study is required to explore the exact mechanisms of the protective effect of controlled reoxygenation on hypoxic apoptosis.

Currently, CPB treatment of cyanotic infants is controversial and involves controlled reoxygenation therapy. It is necessary to understand how high initial reoxygenation levels of CPB treatment in cyanotic infants should be, because it impacts clinical decision making. We provide evidence for the protective effect of controlled reoxygenation during hypoxia-induced brain injury. Controlled reoxygenation may provide an effective therapeutic strategy for hypoxia-induced tissue injury via regulation of the JAK2/STAT3 signaling pathway. It will help make better informed clinical treatment decisions for cyanotic infants.

Several limitations in this study should be taken into consideration: first, 24 newborn male and female P3 mice were used in this study, and gender differences may have had an effect on the experimental result. Indeed, before beginning the formal experiment, we considered that sex could be a possible factor that would cause some differences among different individuals. However, we failed to discover the difference between male and female mice based on the results of immunohistochemistry, immunocytochemistry, and western blotting. We suspect that the effects of our hypoxia and reoxygenation treatments are restricted to the respiratory system, but do not affect the blood; thus gender differences are not significant enough to make a substantial difference between individuals. Second, we did not make any attempt to differentiate neurons from glial cells in our study (see Materials and Methods), the conclusion that the observed changes are more pertinent in regard to neurons based on the result of immunochemistry is rather weak, which is a flaw in our experiment and will be improved in our future research. Third, all western blot data refers to whole tissue without any attempt to differentiate neurons from glial cells.

## CONCLUSION

In this study, we revealed that controlled reoxygenation treatment could reduce neuron and glial cell apoptosis caused by hypoxia and exert an anti-inflammation effect on hypoxia-induced brain injury mice by downregulating JAK2/STAT3 and AMPK/mTOR signaling pathway. Our findings may propose a potential therapeutic strategy of controlled reoxygenation on CPB during CHD.

## MATERIALS AND METHODS

### Reagents

Terminal deoxynucleotidyl transferase-mediated dUTP nick end labeling (TUNEL) assay kits were obtained from Roche Molecular Biochemicals (Mannheim, Germany). BCA protein quantification kit was purchased from Merck Millipore Technology (Darmstadt, Germany). The primary antibodies used were as follows: rabbit anti-goat, goat anti-mouse and goat anti-rabbit secondary antibodies, which were purchased from the Zhongshan Company (Beijing, China).

### Animal study

Newborn male and female post-natal day 3 (P3) mice were purchased from Beijing Vital River Laboratory Animal Technology Co., Ltd. Twenty-four mice were used to establish the animals model in one modeling process, and a total of three modelings were accomplished in this study. All procedures were performed according to the Center for Animal Resources and Development regulations for animal care, and this study was approved by the ethics committee of Children's Hospital of Chongqing Medical University, Ministry of Education Key Laboratory of Child Development and Disorders, China. First, the mice were housed under a controlled temperature (23–25°C and 55% humidity) and allowed free access to food and water. A 12 h light-dark cycle was maintained with lights on between 8 a.m. and 8 p.m. The mice pups with their mother were randomly transferred into a gas chamber filled with 10% O_2_ and cages (six post-natal mice per cage) and without changes of accessories – no mice were subjected to reoxygenation prematurely. After 8 days of hypoxia, standard reoxygenation was performed in three groups at the following concentrations: 21% O_2_, 50% O_2_, and 100% O_2_ for 24 h, respectively. In the case of controlled reoxygenation, the reoxygenation step was performed with 14% O_2_ for 4 h, following 18% O_2_ for 8 h, and finally 21% O_2_ for 12 h. The treatments lasted 24 h for all groups. A total of 24 post-natal mice were selected and euthanized, and the brain tissue was used for the following experiments.

### Immunohistochemistry and immunocytochemistry

Immunohistochemistry and immunocytochemistry experiments were carried out on P13 mice pups. The brain tissue was fixed with 4% paraformaldehyde and embedded in Tissue-Tek OCT compound (Miles), frozen on dry ice and stored at −80°C. 6 µm cryostat sections of brain tissue were prepared for immunohistochemical analysis. The apoptotic cells were detected by terminal deoxynucleotidyl transferase-mediated dUTP nick end labeling (TUNEL) assay kit (Roche Molecular Biochemicals, Mannheim, Germany). In brief, the tissue was fixed by 4% formalin for 24 h, and we then performed TUNEL staining according to the manufacturer's instructions. The index of apoptosis was expressed by the number of TUNEL-positively stained apoptotic neurons and glial cells/the total number of neurons and glial cells counted×100%. The caspase-3 positive cells and expression of Iba-1 were immunohistochemically analyzed. Peroxidase activity was blocked with 10% H_2_O_2_ and the sections were treated with citrate buffer (pH 6.0, at 96°C for 30 min), cooled for 20 min, and incubated in a blocking buffer [composed of 20% serum, 0.2% Triton-X-100 in phosphate buffer saline (PBS)] for 1 h at room temperature. The sections were incubated overnight with anti-Iba-1 antibody (ab48004, Abcam, Cambridge, UK), and the immunolabeling was visualized using the Vectastain Universal Elite ABC Kit.

### Western blotting

The extracted protein was washed with PBS and then lysed with Laemmli sample buffer. Protein concentration was measured by using BCA Protein Assay Kit (Thermo Fisher Scientific, Waltham, USA). Protein samples of 15 μg were subjected to SDS-PAGE electrophoresis and separated proteins were transferred to polyvinylidene difluoride (PVDF) membrane (Immobilon-P; EMD Millipore, Darmstadt, Germany). The membrane was blocked with 5% of skimmed milk in TBS-T for 2 h and then incubated with the primary antibodies against cleaved caspase-3 (#9661, Cell Signaling Technology, USA), cleaved PARP (194C1439, Santa Cruz Biotechnology, CA, USA), LCN2 (#44058, Cell Signaling Technology), Iba-1 (ab48004, Abcam), GFAP (#12389, Cell Signaling Technology), JAK2 (#3230, Cell Signaling Technology), p-JAK2 (#4406, Cell Signaling Technology), STAT3 (#4904, Cell Signaling Technology), p-STAT3 (#9134, Cell Signaling Technology), BNIP3 (#3769, Cell Signaling Technology), p-AMPK (#2531, Cell Signaling Technology), AMPK (#5831, Cell Signaling Technology), p-mTOR (#5536, Cell Signaling Technology) and mTOR (#2983, Cell Signaling Technology), overnight at 4°C. After washing three times in Tris Buffered Saline Tween (TBST), the membrane was incubated with secondary antibody at room temperature for 1 h. As an internal control, expression of β-actin was measured by using anti-β-actin antibody as primary antibody and HRP-conjugated anti-rabbit antibody as secondary antibody. Protein bands were detected by using Luminata Forte Western HRP Substrate (Millipore, Billerica, MA, USA) with a Bio-Rad ChemiDox XRS+ imaging system (Bio-Rad, Hercules, CA, USA). What needs illustration is that all western blotting data refers to whole tissue without any attempt to differentiate neurons from glial cells.

### RNA isolation and cDNA generation

Total RNA from stimulated tissue was isolated by RNeasy Micro Kit (Qiagen, Germany) according to the manufacturer's instructions. Complementary DNAs (cDNAs) were prepared from RNA using SuperScript III Reverse Transcriptase (RT) (Invitrogen, Gaithersburg, MD, USA). RT reaction was carried out at 50°C for 1 h, and then one unit of RNase H (Takara, Shiga, Japan) was treated at 37°C for 15 min.

### Quantitative PCR

The transcript of LCN2, was measured by SYBR Green PCR Master Mix (Applied Biosystems) with the following primer sets on ABIViiA7 Real-Time PCR System. Expression of β-actin was used as internal control for analysis. The primer sequences (5′→3′) were as follows:

LCN2, Forward: TCGCTACTGGATCAGAACATTTG; Reverse: GAACTGGTTGTAGTCCGTGGTG; β-actin, Forward: CTTTGCAGCTCCTTCGTTGC; Reverse: CCTTCTGACCCATTCCCACC.

### Measurement of cytokine and chemokine

Cytokine IL-6, cytokine TNF-α and chemokine CXCL10 were measured by ELISA kit (R&D Systems; Minneapolis MN, USA) according to the manufacturer's instructions. In brief, the tissue lysate was incubated for 2 h at room temperature in 96-well plates coated with anti-IL-6, anti-TNF-α or anti-CXCL10 antibody. After washing, the capture antibody was added and incubated for 2 h at room temperature. The reaction was measured by the absorbance at 450 nm.

### Statistical analysis

All data were presented as means±standard error of the mean (s.e.m.). Differences were compared by Analysis of Variance (ANOVA) followed by Bonferroni correction for post-hoc *t*-test, where appropriate. Probabilities of <0.05 were considered to be statistically significant. All of the statistical analyses were performed with the GraphPad Prism software version 5.0 (GraphPad Software, San Diego, CA, USA).
